# Association of emergence of new mutations in circulating tumuor DNA during chemotherapy with clinical outcome in metastatic colorectal cancer

**DOI:** 10.1186/s12885-021-08309-2

**Published:** 2021-07-22

**Authors:** Ning Jia, Lianpeng Chang, Xin Gao, Xiaohua Shi, Xuelin Dou, Mei Guan, Yajuan Shao, Ningning Li, Yuejuan Cheng, Hongyan Ying, Zhao Sun, Yanping Zhou, Lin Zhao, Jianfeng Zhou, Chunmei Bai

**Affiliations:** 1 Department of Medical Oncology, Peking Union Medical College Hospital, Chinese Academy of Medical Sciences and Peking Union Medical College, 1 Shuaifuyuan, Dongcheng District, Beijing, 100730 China; 2 Geneplus-Beijing, Beijing, China; 3 Department of Radiology, Peking Union Medical College Hospital, Chinese Academy of Medical Sciences and Peking Union Medical College, Beijing, China; 4 Department of Pathology, Peking Union Medical College Hospital, Chinese Academy of Medical Sciences and Peking Union Medical College, Beijing, China

**Keywords:** New mutation, Circulating tumour DNA, Next generation sequencing, Biomarker, Metastatic colorectal cancer

## Abstract

**Background:**

The understanding of molecular changes in mCRC during treatment could be used to personalise therapeutic strategies. The aim of our study was to explore the association of circulating tumour DNA (ctDNA) with clinical outcome in metastatic colorectal cancer (mCRC).

**Methods:**

Sequential patients with mCRC receiving standard first-line chemotherapy were included prospectively. Both plasma ctDNA and serum CEA were assessed in samples obtained before treatment and after 4 cycles of chemotherapy (C4). Computed tomography (CT) scans were carried out at baseline and post-C4 (8–10 weeks) and were assessed using Response Evaluation Criteria In Solid Tumours version 1.1 (RECIST v1.1). Target-capture deep sequencing with a panel covering 1021 genes was performed to detected somatic mutations in ctDNA.

**Results:**

A total of 20 patients were prospectively included and treated with either leucovorin, fluorouracil, and oxaliplatin (FOLFOX) (15/20) or leucovorin, fluorouracil, and irinotecan (FOLFIRI) (5/20). Median follow-up was 6.9 months (range 1.6–26.6). Somatic mutations for baseline ctDNA analysis were identified in 85% (17/20) of the patients. Mutation variations of ctDNA after chemotherapy were tested in 16/20 (80.0%) of the patients. In multivariate analyses, a high baseline molecular tumour burden index (mTBI) in ctDNA was associated with a higher risk of disease progression, as well as emergence of new mutations in ctDNA during chemotherapy. Patients with newly detected mutations had shorter progression-free survival (PFS) compared to those without (median 3.0 versus 7.3 months; hazard ratio (HR), 5.97; 95% confidence interval (CI), 0.70–50.69; *P* = 0.0003). Fold changes in mTBI from baseline to post-C4 were obtained in 80.0% (16/20) of the patients, which were also related to PFS. Patients with fold reduction in mTBI above 0.8-fold had longer PFS compared to those below (median 9.3 versus 4.1 months; HR, 4.51; 95% CI, 1.29–15.70; *P* = 0.0008).

**Conclusions:**

Newly detected mutations in ctDNA during treatment might potentially be associated with clinical outcome in mCRC and may provide important clinical information.

**Supplementary Information:**

The online version contains supplementary material available at 10.1186/s12885-021-08309-2.

## Introduction

The identification of prognostic biomarkers able to guide treatment decision remains challenging in the management of metastatic colorectal cancer (mCRC) patients. A prognostic marker would help personalise therapeutic strategies. Personalising treatment according to biomarkers might have important clinical utility and lead to improved therapeutic outcome. Therefore, it is crucial to identify prognostic biomarkers in the management of mCRC.

The pattern of genetic alterations in mCRC is a dynamic model affected by several intrinsic and extrinsic factors, including tumour genetic instability and treatment pressure, which is generally assessed in bioptic samples. However, tissue from biopsy material can sometimes be limited, difficult to access or yield poor quality DNA. Additionally, a biopsy can only cover a limited area of the tumour tissue and only represents a glimpse of the tumoural molecular characterisation. In this consideration, circulating tumour DNA (ctDNA) carrying tumour-specific genomic alterations in the peripheral blood can offer significant advantages given that repeatability of blood draw can be performed with relative ease, making it a potential alternative to onerous repeat biopsies [1, 2]. The dynamisms and the heterogeneity of tumours may be detected using this method, which allows for real-time evaluation.

Several studies have demonstrated the prognostic value of identification of mutations in mCRC [3–7], and some studies have demonstrated that emerging new mutations could be detected in ctDNA [8–10]. CtDNA may be used to track clonal evolution and targeted drug responses in mCRC patients [11]. Although the detection of KRAS mutations in ctDNA may be of prognostic value [12, 13], the clinical utility of newly detected mutations in ctDNA has been insufficiently explored. Moreover, the use of next generation sequencing (NGS)-based techniques might significantly improve the identification of genomic alterations in patients with mCRC.

In this study, by analysis of somatic mutations with a panel covering 1021 genes using the NGS method, we explored the potential association of the detection of new mutations in ctDNA during chemotherapy with poor prognosis in the patients with metastatic colorectal cancer.

## Methods

### Study design

This prospective study recruited patients from Peking Union Medical College Hospital. Eligible patients had Response Evaluation Criteria In Solid Tumours version1.1 (RECIST v1.1) measurable, chemotherapy naïve mCRC, and were to receive standard first-line combination chemotherapy (leucovorin, fluorouracil, and oxaliplatin (FOLFOX) or leucovorin, fluorouracil, and irinotecan (FOLFIRI) with or without targeted therapy. Treatment continued until the establishment of progressive disease (PD) or until the completion of 12 cycles of treatment followed by maintenance with capecitabine alone when the disease was considered complete response (CR), partial response (PR) or stable disease (SD) [14].

Both plasma ctDNA and serum carcinoembryonic antigen (CEA) were assessed in samples obtained before treatment and after 4 cycles of treatment (C4). Computed Tomography (CT) scan of the chest, abdomen and pelvis was performed at baseline and after 4 cycles of chemotherapy (usually 8–10 weeks after starting treatment). These scans were assessed by a single radiologist, and disease response was evaluated as CR, PR, SD or PD according to RECIST v1.1 [14].

We defined right-sided colon cancer as cancer of the cecum and the ascending colon up to the hepatic flexure and the transverse colon. Left-sided colon cancer comprises cancer of the splenic flexure and cancer in regions distal to the splenic flexure, including the rectum.

### Ethics statement

This study was approved by the Medical Ethics Committee of Peking Union Medical College Hospital (ZS-1358) and carried out in accordance with the Helsinki Declaration on experimentation involving human subjects. Twenty patients with pathologically confirmed mCRC in our department at Peking Union Medical Hospital were recruited. All patients signed informed consent.

### Sample preparation, storage and DNA extraction

Peripheral blood was collected in ethylenediaminetetraacetic acid (EDTA) tubes and centrifuged for 10 min at 1600 g at 4 °C within 2 h of collection. The cell pellets containing peripheral blood lymphocytes (PBLs) were stored at − 20 °C until further use. The supernatants were further centrifuged at 16,000 g for 10 min, and plasma was harvested and stored at − 80 °C until needed. Circulating DNA (cDNA) was extracted from plasma samples with the QIAamp Circulating Nucleic Acid Kit (Qiagen, Hilden, Germany). Genomic DNA (gDNA) were extracted from peripheral blood cells using the QIAamp DNA Blood Mini Kit (Qiagen, Hilden, Germany). Both DNA extractions were performed according to the manufacturer’s instructions. gDNA was sequenced as the normal control sample [15].

### Target capture and NGS

Sequencing libraries of both cDNA and gDNA were constructed with the KAPA DNA Library Preparation Kit (Kapa Biosystems, Wilmington, MA, USA) according to the manufacturer’s protocol. Libraries were hybridised to custom-designed biotinylated oligonucleotide probes (Integrated DNA Technologies, Iowa, IA, USA). Capture probe was designed to cover hot exons or hot regions of 1021 genes frequently mutated in solid tumours (gene list in Table S1). DNA sequencing was performed using the HiSeq 3000 Sequencing System (Illumina, San Diego, CA) with 2 × 101-bp paired-end reads.

### Sequencing data analysis

From raw data, terminal adaptor sequences and low-quality reads were removed. BWA (version 0.7.12-r1039) was employed to align the clean reads to the reference human genome (hg19). Picard (version 1.98) was used to mark polymerase chain reaction (PCR) duplicates. Realignment and recalibration was performed using GATK (version 3.4-46-gbc02625). Single nucleotide variants (SNVs) were called using MuTect (version 1.1.4) and NChot, a software developed in-house to review hotspot variants [16]. Small insertions and deletions (indels) were identified by GATK. Somatic copy number alterations were identified with CONTRA (v2.0.8). Significant copy number variation was expressed as the ratio of adjusted depth between ctDNA and control gDNA. The final candidate variants were all manually verified in the Integrative Genomics Viewer (IGV).

### Clonal population structure construction and molecular tumour burden index (mTBI)

PyClone was used to analyse the clonal population structure of ctDNA before treatment and to cluster trunk mutations in each ctDNA collected serially from each patient. mTBI was calculated as the mean allele fraction of trunk mutations in the mutation cluster in each ctDNA [17].

### Statistical analysis

Descriptive statistics and the Mann–Whitney U-test were used to assess the clinical and biochemical variables associated with baseline mTBI. Cox proportional hazards analysis (enter method) was used to estimate the hazard ratios with 95% confidence intervals for the treatment effect in relation to progression-free survival (PFS) and biomarkers or prognostic clinical information. Multivariate analyses were adjusted for well-characterised risk factors: age, gender, Eastern Cooperative Oncology Group (ECOG) performance status (PS), location of primary tumour and metastatic synchronicity. None of these variables was missing. Correlations between the circulating biomarkers (ctDNA and CEA) and tumour response (measured as per RECIST1.1) were assessed using Spearman’s rank correlation. The Mann–Whitney U-test was also used to assess whether there was a significant difference in PFS between groups with and without new mutations in ctDNA. PFS was analysed using the Kaplan–Meier method and compared with the log-rank test. PFS was defined as the time elapsed from the first cycle of treatment until the date of first progression or death (all causes) or censoring at last follow-up. Receiver operating characteristic (ROC) analysis was performed to evaluate the ability of variables to predict PFS and different response. The cut-off values were estimated at various sensitivities and specificities and were determined at the maximum Youden’s index. The Mann–Whitney U-test was used to assess whether there was a significant difference in quantitative variables between groups of different responses at first restaging. For qualitative variables, patients were grouped according to the presence or absence of each variable, and Fisher’s exact was used to compare variables in relation to tumour response. All statistical analyses were performed with SPSS (v.22.0; STATA, College Station, TX, USA) or GraphPad Prism (v. 7.0; GraphPad Software, La Jolla, CA, USA) software. Statistical significance was defined as a two-sided *P*-value of < 0.05.

## Results

### Patient characteristics

**Fig. 1:**
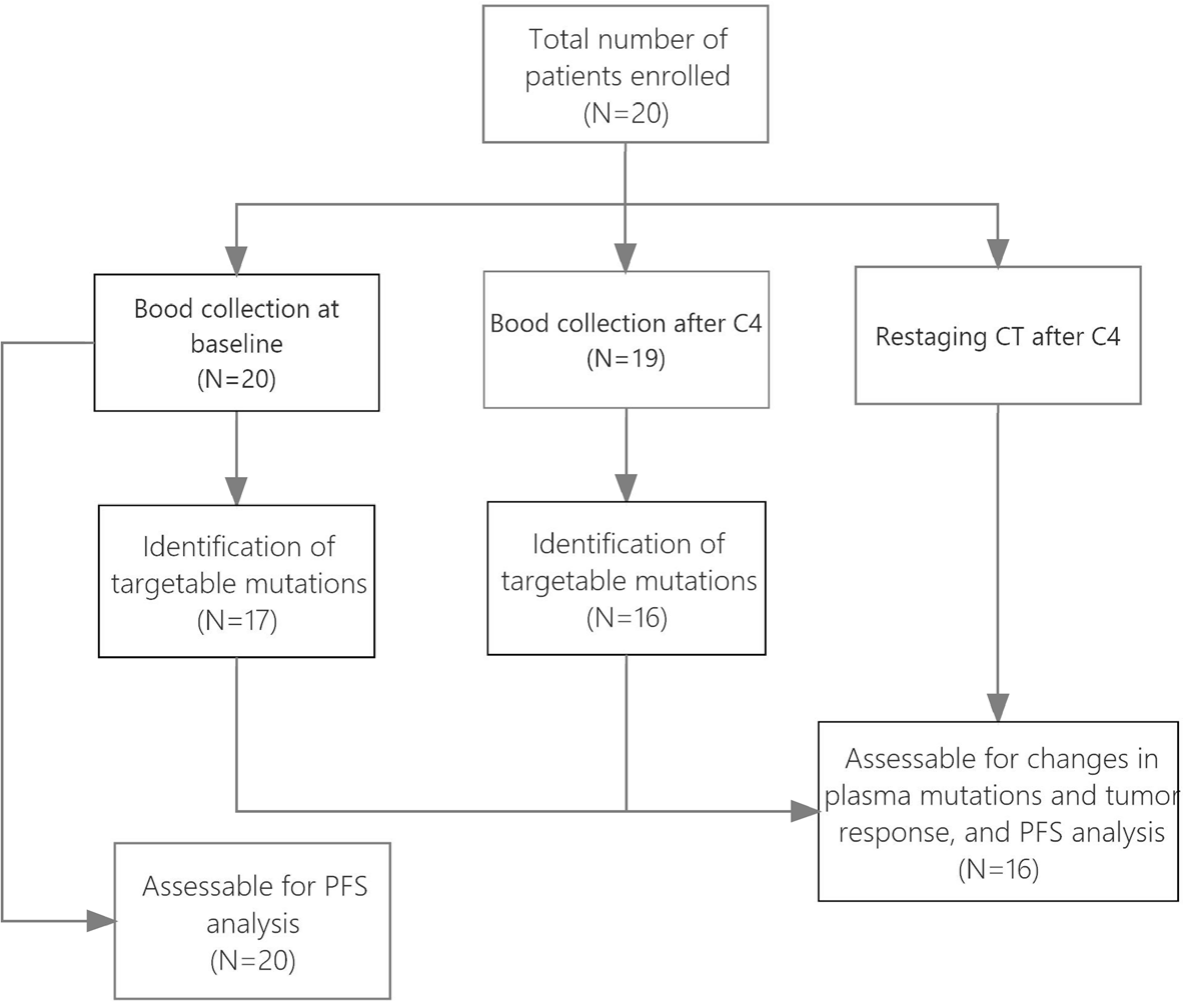
Diagram showing the flow of patients through the study, including the number of patients included in each of the analysis end points

Twenty patients were prospectively enrolled, all of whom had at least 1 baseline blood draw. None of the patients with RAS wild type in this cohort could afford the expense of additional anti-EGFR monoclonal antibodies that were not covered by insurance back at that time. Figure 1 summarises the flow of patients through the study. At the time of analysis, 17 of the 20 (85.0%) patients evaluable for PFS had experienced disease progression, providing a median PFS of 7.3 months (interquartile range (IQR): 4.1–9.9 months). The characteristics of the patients and association with baseline mTBI are shown in Table 1. The mTBI at baseline was significantly higher for male patients versus female patients (*P* = 0.0100). No significant difference in baseline mTBI was associated with the other patient characteristics tested. The trunk mutations clustered in each ctDNA and its variations after treatment are listed in Table S2.

**Table 1:** Patient characteristics and association with baseline mTBI (*N* = 20)

Characteristic	N (%)	Baseline mTBI (%) Median (IQR)	*P* Value
All patients	20		
Age (years), median	60 (34–76)		
Age group			
< 60	11 (55.0)	3.62 (0.90–27.54)	–
60+	9 (45.0)	23.79 (4.11–48.52)	0.2601
Gender			
Female	6 (30.0)	3.24 (0.00–4.11)	–
Male	14 (70.0)	38.72 (6.80–57.99)	0.0111
ECOG			
0	15 (75.0)	5.00 (1.24–38.72)	–
1	5 (25.0)	45.52 (6.37–73.77)	0.0978
Primary tumor site			
Left-side colon	16 (80.0)	9.01 (1.38–44.57)	–
Right-side colon	4 (20.0)	13.71 (3.34–60.85)	0.8198
Synchronicity of metastasis			
Metachrone	10 (50.0)	12.49 (1.65–45.9)	–
Synchrone	10 (50.0)	7.20 (2.43–46.3)	1.0000
Primary tumor(s)			
Resected	15 (75.0)	5.00 (1.79–38.72)	–
Unresected	5 (25.0)	41.70 (4.70–59.36)	0.3941
Metastatic site(s)			
1 without peritoneum	9 (45.0)	9.40 (2.24–47.02)	–
2+ without peritoneum	11 (55.0)	8.62 (1.79–38.72)	0.8817
Peritoneum	0		–
Baseline serum CEA			
Normal	5 (25.0)	1.79 (0.62–13.52)	–
Elevated	15 (75.0)	16.36 (4.11–48.52)	0.0522
Chemotherapy regimen			
FOLFOX	15 (75.0)	8.62 (3.24–45.52)	–
FOLFIRI	5 (25.0)	16.36 (1.52–51.94)	0.9327
Bevacizumab received			
Yes	1 (5.0)	23.79	–
No	19 (95.0)	8.62 (1.79–45.52)	–

*P* value indicates a significance level of < 0.05

*IQR* interquartile range, *ECOG* Eastern Cooperative Oncology Group performance status, *CEA* carcinoembryonic antigen

### Detection of ctDNA at baseline and during treatment

At least 1 mutation was identified in the baseline plasma of 85.0% (17/20) cases. Genomic mutations of ctDNA observed twice or more in the overall cohort at baseline are shown in Fig. S1. There were 40 mutated genes detected in total. The 3 most frequently mutated genes were TP53 (94.1%, 16/17), APC (70.6%, 12/17) and KRAS (47.1%, 8/17). No regularity of mutation distribution in patients with different age, gender, ECOG PS and location of primary tumour was seen. Blood samples after C4 from 19/20 patients were collected. Mutations in plasma ctDNA after C4 were detectable in 16/19 (84.2%) patients, 4 of whom emerged 11 new mutated genes as shown in Table 2. The somatic mutations identified in plasma and the respective abundances of ctDNA mutations and their variations between baseline and after treatment are shown in Table S3.

**Table 2:** New mutations detected in plasma ctDNA during treatment

Patient ID	Time point	New mutation	cHGVS	pHGVS	Function	VAFs (%)
P06	Post-C2	KRAS	c.38G > A	p.G13D	missense	0.36
P07	Post-C4	CDK13	c.604_605insT	p.R202Lfs*68	frameshift	1.10
	Post-C4	MTOR	c.617G > A	p.R206H	missense	0.73
P08	Post-C4	BRCA2	c.6232G > A	p.G2078R	missense	0.90
	Post-C4	MYC	c.1211A > G	p.K404R	missense	0.32
	Post-C4	FGFR2	c.269A > T	p.E90V	missense	0.56
	Post-C4	EGFR	c.2327G > A	p.R776H	missense	0.25
P10	Post-C4	PIK3R2	c.451C > T	p.P151S	missense	1.23
	Post-C4	DNMT3A	c.2077C > T	p.R693C	missense	0.57
	Post-C4	NTRK1	c.1859G > A	p.C620Y	missense	0.60
	Post-C4	MLL	c.10480A > G	p.N3494D	missense	0.60

*cHGVS* Coding DNA reference sequences (Human Genome Variation Society), *pHGVS* Protein level amino acid sequences (Human Genome Variation Society), *Post-C2* After 2 cycles of chemotherapy, *Post-C4* After 4 cycles of chemotherapy

### Concordance of KRAS and BRAF V600E mutation between tumour tissue and matched plasma ctDNA samples

Nine of the 20 enrolled patients had results of tumoural KRAS status determined by NGS platform-ion torrent PGM and/or amplification refractory mutation system (ARMS). Six of the 20 patients had results of tumoral BRAF V600E status determined by NGS platform-ion torrent PGM and/or real-time polymerase chain reaction (RT-PCR). The KRAS and BRAF V600E status in the matched plasma and tissue from each patient is summarised in Table S4, which was concordant in every case.

### Variations in ctDNA during treatment and tumour response

At the time of analysis, 8 of the 20 patients in this study evaluable for tumour response had partial response with a 40.0% objective response rate (ORR), including 5 of the 16 patients evaluable for new mutations with a 31.0% ORR. There was a statistically significant difference in fold change in mTBI after C4 between groups of patients with tumour response and non-response (*P* = 0.0085, Fig. S2A). However, no significant difference was seen in fold change in serum CEA (*P* = 0.0687, Fig. S2B). Relations between tumour response after C4 and clinical factors are shown in Table 3. Fold change in mTBI after C4 was related to tumour response (Mann–Whitney U-test at *P* = 0.0076). Tumour response was not associated with the other patient characteristics tested. In the 14 patients with reduction of mTBI, the partial response rate and stable disease rate were 35.7% (5/14) and 50.0% (7/14), respectively. Two patients with progressive disease emerged new mutations in ctDNA during treatment. During the follow-up period, we collected the blood samples of P03 and P04 at the time of disease progression. The mTBI of P03 reduced from 48.6% at baseline to 0.0% at restaging after C4 with achievement of a partial response, which increased to 43.3% at the time of disease progression. The mTBI of P04 showed a similar variation tendency to P03: 45.5% at baseline, 2.1% at restaging with stable disease and 42.2% at the time of disease progression. The objective response rate was 41.7% (5/12) and 0.0% (0/4) in the patients without and with emergence of new mutations in ctDNA during treatment, respectively, while there was no statistically significant difference in ORR between patients with and without emergence of new mutations in ctDNA during treatment (Fisher’s exact at *P* = 0.0885).

**Table 3:** Relations between tumor response and clinical factors

Variable	*P* value
Age	0.3338
Gender	0.2615
ECOG	1.0000
Location	1.0000
Synchronicity	0.5962
Fold change in CEA	0.0687
Fold change in mTBI	0.0076
New mutations	0.0885

### Baseline ctDNA and PFS

**Fig. 2:**
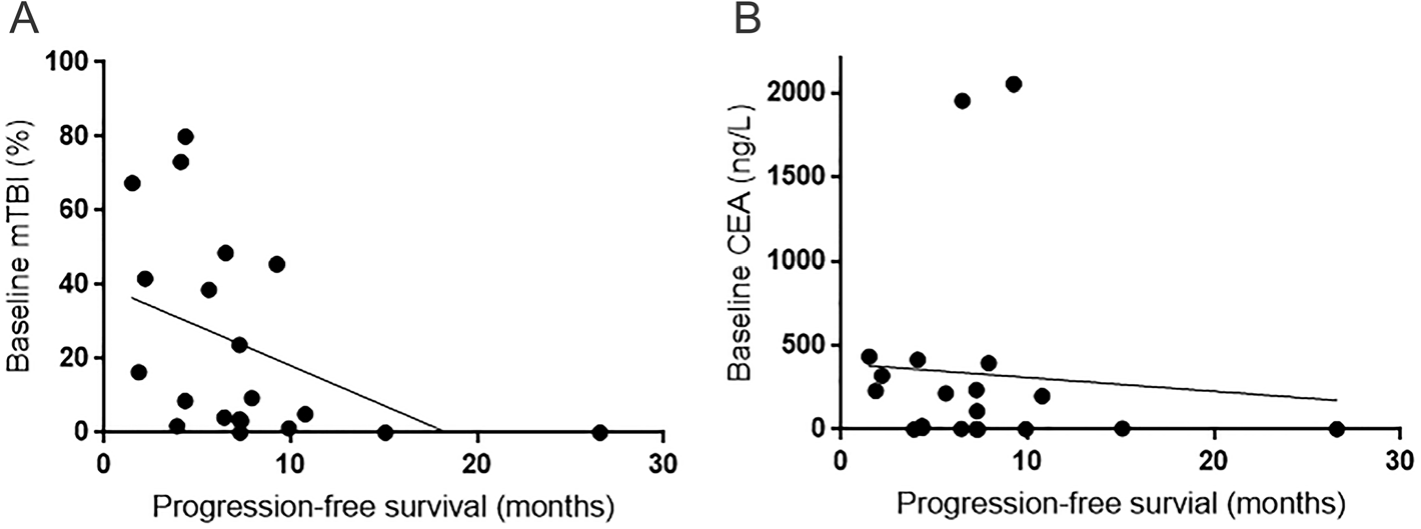
Correlations between baseline mTBI and CEA level and PFS. **a** Negative correlation between the baseline mTBI level and PFS was seen: Spearman correlation at *P* = 0.0083, *r* = − 0.5725. **b** No correlation between the baseline serum CEA level and PFS was observed: Spearman correlation at *P* = 0.2928, *r* = − 0.2475

**Fig. 3:**
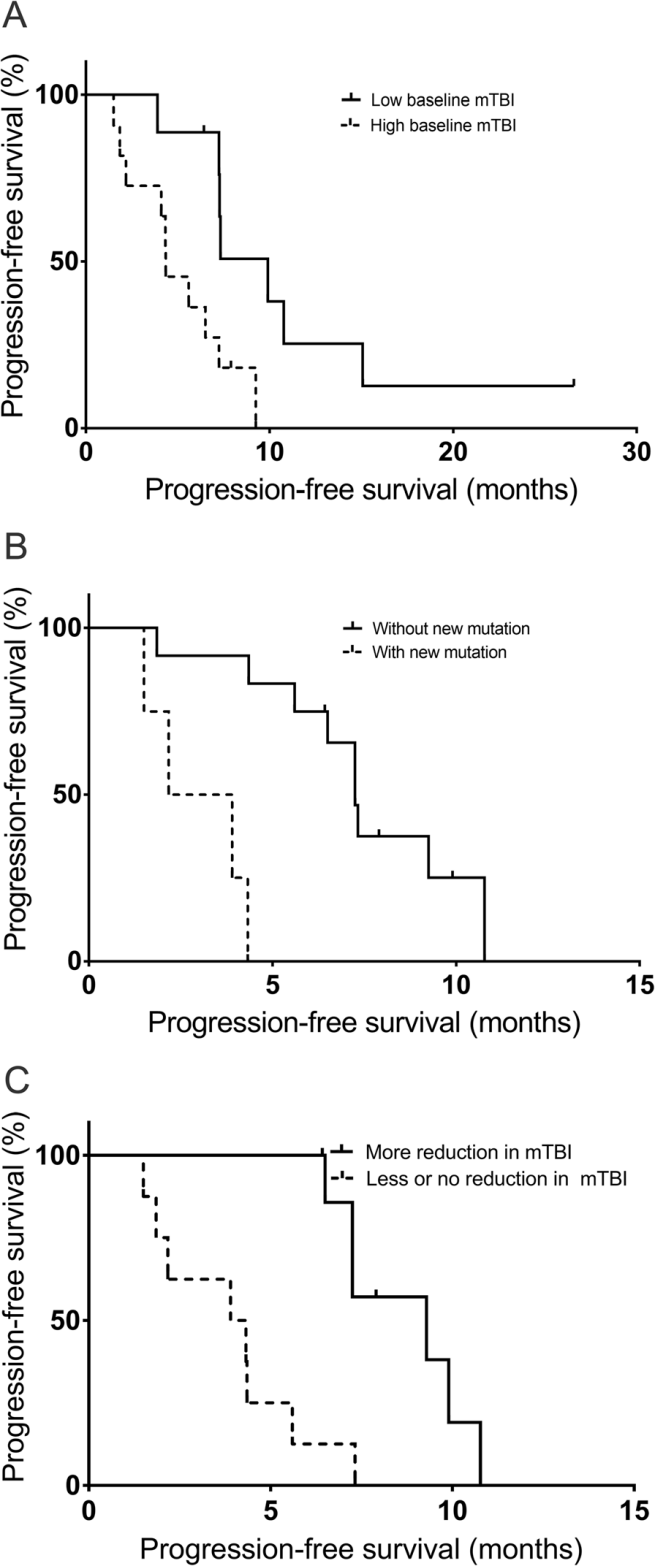
Kaplan–Meier survival plots of progression-free survival (PFS) in different patient groups in this cohort. **a** Patients with baseline mTBI level below 6.81% had longer PFS compared to those above (*P* = 0.0115). **b** Patients with identification of new mutations during treatment had a significant shorter PFS than the patients without identification of new mutations (*P* = 0.0003). **c** Patients with fold reduction in mTBI above 0.8-fold had longer PFS compared to those below (*P* = 0.0115)

Predictive value of clinical factors, including age, gender, ECOG PS, location of primary tumour, synchronicity of metastasis, serum CEA at baseline, mTBI at baseline and identification of new mutations during chemotherapy was evaluated. In univariate analyses, we observed that the high mTBI level at baseline recorded as a continuous variable was significantly associated with a shorter PFS (*P* = 0.0494), as well as for patients with emergence of new mutations in ctDNA during treatment versus those without mutations (*P* = 0.008). In multivariate analyses, the high mTBI level at baseline remained significantly associated with a shorter PFS (*P* = 0.0084), as well as for male patients versus female patients (*P* = 0.0204), patients with ECOG PS 1 versus ECOG PS 0 (*P* = 0.0455), patients with synchronous versus metachronous metastatic disease (*P* = 0.0423) and patients with emergence of new mutations in ctDNA during treatment versus those without (*P* = 0.0033), which are shown in Table 4. Negative correlation between the baseline mTBI and PFS was seen in this cohort of patients (Spearman correlation, *P* = 0.0083, *r* = − 0.5725; Fig. 2a). The optimal baseline mTBI for predicting PFS, as determined by the ROC curves (ROC area = 0.83, *P* = 0.0126), was 6.8%. Patients with baseline mTBI below 6.8% had longer PFS compared to those above (median 9.9 versus 4.35 months; hazard ratio (HR), 2.97; 95% confidence interval (CI), 1.08–8.18; *P* = 0.0115; Fig. 3a). There was no significant association of the baseline serum CEA level with PFS (*P* = 0.7363). No correlation between the baseline serum CEA level and PFS was observed (Spearman correlation, *P* = 0.2928, *r* = − 0.2475; Fig. 2b).

**Table 4:** Univariate and multivariate analyses of factors for progression-free survival using cox regression model (*N* = 16)

	Univariate analysis			Multivariable analysis		
	HR	95.0% CI	*P* value	HR	95.0% CI	*P* value
Age	1.0050	0.9534–1.0593	0.8539	1.0787	0.9595–1.2128	0.2048
Gender	1.0956	0.2897–4.1431	0.8930	784.8123	2.8072–219,412.9720	0.0204
ECOG	0.7963	0.4319–1.4681	0.4655	4.9876	1.0327–24.0880	0.0455
Location	1.0421	0.2696–4.0276	0.9523	11.4735	0.1187–1108.9594	0.2955
Synchronicity	2.9627	0.8893–9.8702	0.0769	54.3070	1.1487–2567.3704	0.0423
Baseline CEA	1.0000	0.9992–1.0007	0.9190	1.0003	0.9983–1.0024	0.7363
Baseline mTBI	1.0236	1.0001–1.0476	0.0494	1.1574	1.0381–1.2903	0.0084
New mutations	0.0486	0.0052–0.4540	0.0080	0.0006	0.0000–0.0831	0.0033

*ECOG* Eastern Cooperative Oncology Group performance status, *CEA* carcinoembryonic antigen, *mTBI* molecular tumour burden index

### Identification of new mutations during treatment and PFS

The patient group with identification of new mutations during treatment had a significantly shorter PFS than the patient group without identification of new mutations (median 3.0 versus 7.3 months; HR, 5.97; 95% CI, 0.70–50.69; *P* = 0.0003; Fig. 3b).

### CtDNA variation and PFS

In univariate analyses, the association of fold reduction of mTBI with PFS was observed (HR, 2.92, 95% CI, 1.17–7.28; *P* = 0.0214). In multivariate analyses, patients who had a more fold reduction of mTBI had a significantly longer PFS than patients with less or no fold reduction of mTBI after adjustment for age, gender, ECOG PS, location of primary tumour and metastatic synchronicity (HR, 27.27, 95% CI, 1.49–498.10; *P* = 0.0257, Table S5). The optimal fold reduction in mTBI for predicting PFS, as determined by the ROC curves (ROC area = 0.8438, *P* = 0.0209), was 0.8-fold. Patients with fold reduction in mTBI above 0.8-fold had longer PFS compared to those below (median 9.3 versus 4.1 months; HR, 4.51; 95% CI: 1.29–15.70; *P* = 0.0008, Fig. 3c). In contrast, serum CEA level variation did not correlate with PFS.

## Discussion

Our study shows that ctDNA can be identified in a high proportion of patients with treatment-naive mCRC. The evaluation of a panel of 1021 genes allowed us to identify mutations in the plasma at baseline of 17 cases (85% of all 20 patients). Published studies of ctDNA in the patients with mCRC have used varying analytical methods or targeted gene panels which can make data interpretation and comparison across studies difficult. In previous studies, the rate of ctDNA detected in the plasma varied from 73.7 to 90.6% of patients with mCRC [1, 18–21]. Due to constant improvements in sensitivity of technologies, including those to detect somatic changes not identified by the current approach, the rate of detection might increase continuously, which could make ctDNA more applicable in clinical practice.

Our study also demonstrates the high concordance of ctDNA and tumour tissues for the molecular characterisation (KRAS and BRAF status), which was shown in many previous studies [1, 20, 22–24]. As serial biopsies in mCRC are not feasible, ctDNA which appears to be a potential surrogate of both tumour molecular profile and its heterogeneity, might overcome this limitation in routine clinical practice.

Truncal mutations that represent the majority of tumour lesions in a patient are potentially the best candidates for monitoring tumour burden using ctDNA [25]. In our study, both mTBI at baseline and fold change in mTBI, which was calculated on truncal mutations in ctDNA, are associated with PFS in patients with mCRC. The patients with higher mTBI at baseline in this cohort were nearly 3 times more likely to experience disease progression than the patients with lower mTBI. The patients with reduction in mTBI after treatment had a 78% lower risk of disease progression than the patients without. Our study shows the potential of using plasma DNA for molecular stratification, which still need to be confirmed in a larger cohort of patients.

Achievement of response to treatment is one of the aims of anti-tumour therapy. Our data showed that fold change in ctDNA after C4 was related to tumour response. Inexplicably, 2 patients with reduction in ctDNA after 4 cycles of treatment experienced disease progression instead of response to therapy. Subsequently, the ctDNA of the other 2 patients with disease control at first restaging reduced after C4, and rose up at the time of disease progression during the follow-up period. According to the high concordance of variation tendency of ctDNA and response evaluation in these 2 patients, we proposed that the reduction in ctDNA in the 2 patients with disease progression at the first restaging could have reached its nadir before the first restaging and then increased but remained at a lower level than the baseline value at the first restaging, resulting in the illogical mismatch between the reduction in ctDNA and tumour response. Therefore, serial ctDNA monitoring at more time-points might have better accuracy to predict radiological tumour response which need to be validated in further studies.

Our study on mCRC suggests that the detection of new mutations in circulating tumour DNA during treatment may be associated with shorter PFS. The patients with identification of new mutations in this cohort were nearly 6 times more likely to experience disease progression than the patients without, none of whom had tumour response at first restaging. Detection of free-circulating tumour-associated DNA may help the evaluation of colorectal cancer prognosis [12]. Mutational processes evolve across a cancer’s lifespan. Although the dynamics of somatic evolution of CRC remain unclear, those somatic mutations conferring a selective advantage on the cell drive successive waves of clonal expansion with therapeutic resistance, which may correlate with cancer progression. Some mutations in tumour tissue have been validated as prognostic biomarkers in CRC. A negative prognostic effect of gene mutation has been confirmed in a large pooled analysis from the PETACC8 and N0147 trials, where KRAS exon 2 and BRAF mutations were identified as independent predictors of shorter time to recurrence and overall survival (OS) among patients with stage III microsatellite stable (MSS) colorectal cancer [26]. In a meta-analysis investigating the impact of KRAS mutation on outcomes in mCRC patients undergoing liver resection, KRAS mutations were negatively associated with OS (HR = 2.24) and relapse-free survival (HR = 1.89) [3]. In multiple previous studies, BRAF mutation has gained a prominent role as a negative prognostic factor since mCRC patients with BRAF mutation showed shorter median OS [4, 6, 7]. Because of the high concordance of ctDNA and tumour tissues for somatic mutations in the patients with mCRC, mutational evolution in ctDNA may be associated with the prognosis of mCRC. Furthermore, the REVERCE study showed emerging new gene alterations in ctDNA associated with worse survival outcomes [27], which was also shown in this study.

The need for improved molecular stratification of mCRC is important as the identification of the most effective treatment for an individual patient is still mainly based on clinical considerations, such as symptoms, performance status, extent of disease, patients’ preferences and treatment history, while the identification of predictive and/or prognostic biomarkers able to guide treatment decision still stands as a challenging issue in the management of mCRC patients.

It is important to understand that molecular profiles are dynamic and might change under treatment pressure. The development of liquid biopsies makes us able to molecularly monitor our patients in real time and to identify molecular mechanism of drug resistance informing us of possible novel treatment options. Our data suggest that patients with high risk of disease progression might potentially be distinguished by the analysis of ctDNA. However, this is a single-center exploratory study with a small sample size. Our data are insufficient to draw robust conclusions. Further work is needed firstly to better understand the mechanisms of association between ctDNA and clinical outcome and secondly to confirm these preliminary results.

## Supplementary Information

**Additional file 1 **: **Table S1**. Gene list of 1021 gene panel.

**Additional file 2 **: **Table S2**. Abundance of trunk clone and its variations between baseline and after 4 cycles of chemotherapy.

**Additional file 3 **: **Supplementary Fig. S1**. Landscape of genomic mutations in ctDNA observed twice or more in the overall cohort at baseline. Each column represents an individual patient. The top panel shows the molecular tumor burden. The middle panel presents the frequently mutated genes observed twice or more in our cohort study, and the frequency and function are shown on the left and right, respectively. The bottom panel presents patients’ information including age, gender, ECOG, tumor location and stage.

**Additional file 4 **: **Table S3.** Abundances of ctDNA mutations and their variations between baseline and during treatment.

**Additional file 5 **: **Supplementary Fig. S2**. The differences in fold changes in CEA and ctDNA between tumor response and non-response patients. A, Significant difference in fold change in mTBI after C4 between groups of patients with tumor response and non-response was observed: Mann–Whitney U-test at *P* = 0.0085. B, No significant difference was seen in fold change in serum CEA between groups of patients with tumor response and non-response: Mann–Whitney U-test at *P* = 0.0687.

**Additional file 6 **: **Table S4.** Identification of gene status in tumour tissue and baseline ctDNA.

**Additional file 7 **: **Table S5**: Multivariate Cox model assessing the impact on progression free survival of ctDNA variations observed between baseline and post-C4 after adjustment on the following variables: age, gender, Eastern Cooperative Oncology Group Performance Status, location of primary tumor and synchronicity of metastatic disease.

## Data Availability

The authenticity of this article has been validated by uploading the key raw data onto the Research Data Deposit public platform (www.researchdata.org.cn), with the approval RDD number as RDDA2021001965.

## Abbreviations

CtDNA: Circulating tumour DNA
mCRC: Metastatic colorectal cancer
CT: Computed tomography
RECIST: Response evaluation criteria in solid tumours
PD: Progressive disease
CR: Complete response
PR: Partial response
SD: Stable disease
CEA: Carcinoembryonic antigen
C4: Four cycles of treatment
EDTA: Ethylenediaminetetraacetic acid
PBLs: Peripheral blood lymphocytes
cDNA: Circulating DNA
gDNA: Genomic DNA
PCR: Polymerase chain reaction
IGV: Integrative genomics viewer
mTBI: Molecular tumour burden index
PFS: Progression-free survival
ECOG: Eastern cooperative oncology group
PS: Performance status
ROC: Receiver operating characteristic
IQR: Interquartile range
ARMS: Amplification refractory mutation system
RT-PCR: Real-time polymerase chain reaction
ORR: Objective response rate
OS: Overall survival
MSS: Microsatellite stable
