# Supplementary material for: Association of emergence of new mutations in circulating tumuor DNA during chemotherapy with clinical outcome in metastatic colorectal cancer
Source: BMC Cancer. 2021 Jul 22;21:845. doi: 10.1186/s12885-021-08309-2 (PMC8296534; doi:10.1186/s12885-021-08309-2)
Supplement: Supplementary file 1 — Additional file 1 : Table S1. Gene list of 1021 gene panel. [file 12885_2021_8309_MOESM1_ESM.docx]

**Table S1**. Gene list of 1021 gene panel

| ABL1 | ABL2 | ACVR1B | AKT1 | AKT2 | AKT3 | ALK | APC | AR | ARAF |
| --- | --- | --- | --- | --- | --- | --- | --- | --- | --- |
| ARID1A | ARID1B | ARID2 | ASXL1 | ATM | ATR | ATRX | AURKA | AURKB | AXIN1 |
| AXIN2 | AXL | B2M | BAP1 | BARD1 | BCL2 | BCL2L1 | BCOR | BLM | BMPR1A |
| BRAF | BRCA1 | BRCA2 | BRD4 | BRIP1 | BTK | C11orf30 | CASP8 | CBFB | CBL |
| CCND1 | CCND2 | CCND3 | CCNE1 | CD274 | CDC73 | CDH1 | CDK12 | CDK4 | CDK6 |
| CDK8 | CDKN1A | CDKN1B | CDKN2A | CDKN2B | CDKN2C | CEBPA | CHEK1 | CHEK2 | CIC |
| CREBBP | CRKL | CSF1R | CTCF | CTNNA1 | CTNNB1 | CUL3 | CYLD | DAXX | DDR1 |
| DDR2 | DICER1 | DNMT3A | EGFR | ELAC2 | EME2 | EP300 | EPAS1 | EPCAM | EPHA2 |
| EPHA3 | EPHA5 | EPHB2 | EPHB6 | ERBB2 | ERBB3 | ERBB4 | ERCC1 | ERCC3 | ERG |
| ERRFI1 | ESR1 | EXT1 | EXT2 | EZH2 | FAM123B | FAM175A | FANCA | FANCC | FANCD2 |
| FANCG | FANCM | FAS | FAT1 | FAT2 | FBXW7 | FCGR2A | FCGR3A | FGFR1 | FGFR2 |
| FGFR3 | FGFR4 | FH | FLCN | FLT1 | FLT3 | FLT4 | FOXA1 | FOXL2 | FOXP1 |
| FUBP1 | GAB2 | GALNT12 | GATA3 | GNA11 | GNAQ | GNAS | GRIN2A | HDAC1 | HDAC4 |
| HGF | HNF1A | HOXB13 | HRAS | HSP90AA1 | IDH1 | IDH2 | IFNG | IFNGR1 | IGF1R |
| IL7R | INPP4B | IRF2 | IRS2 | JAK1 | JAK2 | JAK3 | KDM5A | KDM5C | KDM6A |
| KDR | KEAP1 | KIT | KRAS | LRP1B | MAP2K1 | MAP2K2 | MAP2K4 | MAP3K1 | MAPK1 |
| MAX | MCL1 | MDM2 | MDM4 | MED12 | MEN1 | MET | MITF | MLH1 | MLH3 |
| MLL | MLL2 | MLL3 | MPL | MRE11A | MS4A1 | MSH2 | MSH3 | MSH6 | MTOR |
| MUTYH | MYC | MYCL1 | MYCN | MYD88 | NBN | NCOR1 | NDUFA13 | NF1 | NF2 |
| NOTCH1 | NOTCH2 | NOTCH3 | NOTCH4 | NPM1 | NRAS | NSD1 | NTHL1 | NTRK1 | NTRK3 |
| PALB2 | PAX5 | PBRM1 | PCK1 | PDCD1LG2 | PDGFRA | PDGFRB | PDK1 | PHF6 | PIK3CA |
| PIK3CB | PIK3CG | PIK3R1 | PIK3R2 | PMS1 | PMS2 | POLD1 | POLE | POT1 | PPM1D |
| PRKAR1A | PTCH1 | PTCH2 | PTEN | PTPN11 | RAD50 | RAD51 | RAD51B | RAD51C | RAD51D |
| RAF1 | RARA | RB1 | RBM10 | RET | RHEB | RHOA | RICTOR | RINT1 | RNASEL |
| RNF43 | ROS1 | RPS6KB1 | RUNX1 | SDHA | SDHAF2 | SDHB | SDHC | SDHD | SERPINB3 |
| SERPINB4 | SETD2 | SLX4 | SMAD2 | SMAD4 | SMARCA4 | SMARCB1 | SMARCE1 | SMO | SOX2 |
| SOX9 | SRC | STAG2 | STAT3 | STK11 | SUFU | SYK | TBX3 | TCF7L2 | TET2 |
| TGFBR2 | TMEM127 | TMPRSS2 | TNFAIP3 | TOP1 | TOP2A | TP53 | TP73 | TSC1 | TSC2 |
| ABCA13 | ABCB1 | ABCC1 | ABCC11 | ABCC2 | ABCG2 | ACACA | ACIN1 | ACTB | ACTG1 |
| ACTG2 | ACVR2A | ACVRL1 | ADAM29 | ADAMTS5 | ADCY1 | AFF1 | AFF2 | AFF3 | AFF4 |
| AHNAK | AKAP9 | ALB | AMOT | ANGPT1 | ANK3 | ANKRD27 | ANKRD30A | ANKRD30B | ANKRD36B |
| APEX1 | APOBEC3B | ARAP3 | ARFGEF1 | ARFGEF2 | ARHGAP26 | ARHGAP29 | ARHGAP35 | ARID4B | ARNT |
| ASCL4 | ASH1L | ASMTL | ASPM | ASTN1 | ASXL2 | ATIC | ATP12A | ATP11B | ATP1A1 |
| ATP2B3 | BAZ2B | BBS9 | BCAS1 | BCL11A | BCL11B | BCL2A1 | BCL2L11 | BCL3 | BCL9 |
| BCLAF1 | BCORL1 | BCR | BIRC2 | BIRC3 | BMPR2 | BNC2 | BPTF | BRD2 | BRD3 |
| BRSK1 | BRWD1 | BTLA | BUB1 | C15orf23 | C15orf55 | C1QA | C1S | C3orf70 | C7orf53 |
| C8orf34 | CACNA1D | CACNA1E | CADM2 | CAMTA1 | CAPN7 | CARD11 | CASP1 | CASQ2 | CBLB |
| CBR1 | CBR3 | CCDC168 | CCNA1 | CCNB3 | CCT3 | CCT5 | CCT6B | CD22 | CD33 |
| CD5L | CDA | CDH11 | CDH18 | CDH23 | CDK13 | CHD1 | CHD1L | CHD3 | CHD4 |
| CHD6 | CHD8 | CHD9 | CHFR | CHI3L1 | CHN1 | CIITA | CKS1B | CLCC1 | CLDN18 |
| CLP1 | CLSPN | CLTC | CNOT3 | CNOT4 | CNTN1 | CNTN5 | CNTNAP1 | CNTNAP5 | COL1A1 |
| COL2A1 | COL5A1 | COL5A2 | COL5A3 | COPS2 | CPS1 | CREB3L1 | CRIPAK | CRLF2 | CRNKL1 |
| CRTC1 | CRYBG3 | CSF1 | CSF3R | CSMD1 | CSMD3 | CSNK1A1 | CSNK1G3 | CSNK2A1 | CTLA4 |
| CTNNA2 | CTNND1 | CUX1 | CYBA | CYP19A1 | CYP1B1 | CYP1A1 | CYP2A13 | CYP2C19 | CYP2C8 |
| CYP2D6 | CYP3A4 | CYP3A5 | DCC | DDX3X | DDX5 | DEK | DHX35 | DHX9 | DIAPH1 |
| DIS3L2 | DLC1 | DMD | DNAH6 | DNAJC11 | DNM2 | DNMT1 | DOCK2 | DOCK7 | DOT1L |
| DPYD | DRGX | DTX1 | DUSP22 | DYSF | EBF1 | ECT2L | EEF1A1 | EGR3 | EIF2AK3 |
| EIF2C3 | EIF3A | EIF4G3 | ELF1 | ELF3 | ELF4 | ELL | ELMO1 | ELN | EMID2 |
| EPC1 | EPHA1 | EPHA4 | EPHA7 | EPHB1 | EPHB4 | EPOR | EPPK1 | EPS15 | ERBB2IP |
| ERCC2 | ESR2 | ETS1 | ETV1 | ETV5 | ETV6 | EWSR1 | EZR | F8 | FAM131B |
| FAM135B | FAM157B | FAM22A | FAM46C | FAM5C | FAP | FASLG | FAT3 | FAT4 | FCGR1A |
| FCGR2B | FCRL4 | FGF10 | FGF14 | FGF23 | FGF3 | FGF4 | FGF6 | FKBP5 | FLG |
| FLI1 | FLNC | FMN2 | FMR1 | FN1 | FNDC4 | FOXA2 | FOXO3 | FOXQ1 | FRG1 |
| FRMPD4 | FUS | FXR1 | FYN | FZD1 | G3BP1 | G3BP2 | GABRA6 | GATA2 | GFRAL |
| GIGYF1 | GKN2 | GLB1L3 | GLI1 | GLI2 | GLI3 | GMPS | GNA12 | GNA13 | GNG2 |
| GPC3 | GPR124 | GPX1 | GRB7 | GRM3 | GSK3B | GSTM5 | GSTP1 | GUSB | H3F3A |
| H3F3C | HCLS1 | HCN1 | HDAC9 | HECW1 | HERC2 | HEY1 | HIP1 | HIST1H1C | HIST1H1D |
| HIST1H1E | HIST1H2AC | HIST1H2AG | HIST1H2AL | HIST1H2AM | HIST1H2BC | HIST1H2BD | HIST1H2BJ | HIST1H2BK | HIST1H2BO |
| HIST1H3B | HIST1H4I | HLF | HMCN1 | HNRPDL | HOXA11 | HOXA13 | HOXA3 | HOXA9 | HOXC13 |
| HOXD11 | HOXD13 | HSD3B1 | HSD3B2 | HSP90AB1 | HSPA8 | HSPD1 | HSPH1 | ICK | IFITM1 |
| IFITM3 | IGF2 | IGF2R | IGLL5 | IGSF10 | IKBKE | IKZF1 | IKZF2 | IKZF3 | IL1RAPL1 |
| IL21R | IL6 | IL6ST | IMPG1 | ING1 | INHBA | INPP4A | INPP5D | INPPL1 | IRF4 |
| IRF6 | ITGB3 | ITK | ITSN1 | JARID2 | KALRN | KAT6A | KAT6B | KCNJ5 | KCNQ2 |
| KDM2B | KDM3B | KEL | KIF5B | KLB | KLF4 | KLHL6 | KLK1 | KRTAP5-5 | L3MBTL1 |
| LAMA2 | LCP1 | LEF1 | LGALS8 | LIFR | LPHN2 | LPP | LRP2 | LRP4 | LRP5 |
| LRP6 | LRRC7 | LRRK2 | LYN | LZTS1 | MACF1 | MAD1L1 | MAGI2 | MAGOH | MAML2 |
| MAML3 | MAP3K13 | MAPK3 | MCC | MCM3 | MDH2 | MECOM | MEF2C | MGA | MIB1 |
| MIOS | MKI67 | MKL1 | MLL4 | MLLT3 | MLLT6 | MMP2 | MMP11 | MN1 | MNDA |
| MNX1 | MPO | MSH4 | MSN | MSR1 | MTHFR | MTRR | MUC5B | MYB | MYBL2 |
| MYH10 | MYH11 | MYH14 | MYH9 | MYO3A | NAP1L1 | NAV3 | NBPF1 | NCAM2 | NCF2 |
| NCF4 | NCK1 | NCOA2 | NCOR2 | NCSTN | NDRG1 | NEB | NFATC4 | NFE2L2 | NFE2L3 |
| NIN | NKX3-1 | NLRC3 | NOD1 | NOS3 | NQO1 | NR1I2 | NR2F2 | NR4A2 | NRP2 |
| NRXN1 | NTM | NTRK2 | NUMA1 | NUP107 | NUP210 | NUP98 | OBSCN | OGDH | OMD |
| OPCML | OR11G2 | OR2T4 | OR4A15 | OR4C6 | OR5L2 | OR6F1 | P2RY8 | P4HB | PABPC1 |
| PABPC3 | PAG1 | PAK1 | PAK3 | PARK2 | PARP1 | PASK | PAX3 | PAX7 | PBX1 |
| PC | PCDH18 | PCLO | PCSK6 | PCSK7 | PDCD1 | PDCD11 | PDE4DIP | PDGFB | PDILT |
| PER1 | PGR | PHF1 | PIK3C2A | PIK3C2B | PIK3C2G | PIK3R3 | PIP5K1A | PKD1L2 | PKHD1 |
| PLAC8 | PLAG1 | PLCB1 | PLCG1 | PLCG2 | PLK1 | PLXNA1 | PLXNB2 | POLQ | POLR2B |
| POM121 | POM121L12 | POTEG | POU2AF1 | PPP1R17 | PPP2R1A | PPP6C | PRAM1 | PRDM1 | PRDM16 |
| PREX2 | PRF1 | PRKAA1 | PRKCB | PRKCI | PRKDC | PRRX1 | PRX | PSG2 | PSIP1 |
| PSMB1 | PSMB5 | PTGS1 | PTGS2 | PTK2 | PTPN13 | PTPN2 | PTPRB | PTPRD | PTPRF |
| PTPRJ | PTPRK | PTPRO | PTPRT | PTPRU | RAB35 | RAC1 | RAC2 | RAD21 | RAD54B |
| RANBP2 | RASA1 | RASGRP1 | RBL1 | RECQL4 | REL | RELN | RFC1 | RGS3 | RHOH |
| RHOT1 | RIT1 | RNF213 | ROBO1 | ROBO2 | ROBO3 | ROCK1 | RPGR | RPL22 | RPTOR |
| RSPO2 | RSPO3 | RUNX1T1 | RUNX2 | RXRA | RYR1 | RYR2 | SBDS | SCUBE2 | SEC31A |
| SEMA3A | SEMA3E | SEMA6A | SERP2 | SERPINA7 | SETBP1 | SETDB1 | SF1 | SF3A1 | SF3A3 |
| SF3B1 | SFPQ | SGCZ | SH3PXD2A | SHH | SI | SIN3A | SLC16A1 | SLC1A2 | SLC22A16 |
| SLC22A18 | SLC22A2 | SLC22A3 | SLCO1B3 | SLIT1 | SLIT2 | SMAD3 | SMC1A | SMC1B | SMURF2 |
| SNCAIP | SNTG1 | SNX29 | SOD2 | SOS1 | SOX10 | SOX17 | SPEN | SPOP | SPRR3 |
| SPSB4 | SPTA1 | SRD5A2 | SRGAP1 | SRGAP3 | SRSF2 | SRSF7 | SSX1 | STAG1 | STAT1 |
| STAT5A | SUCLG1 | SUCLG2 | SULT1A1 | SUZ12 | SVEP1 | SYNCRIP | SYNE1 | TAF1 | TAF15 |
| TAF1L | TAL1 | TBL1XR1 | TBX15 | TBX22 | TCEB1 | TCERG1 | TCF12 | TCF3 | TCF4 |
| TCL1A | TCP11 | TEC | TENM3 | TERT | TFDP1 | TFDP2 | TFE3 | TGFBR1 | TGFBR3 |
| TGM2 | THBS1 | THBS2 | THRAP3 | TJP1 | TLE1 | TLL2 | TLR4 | TLX3 | TMEM132D |
| TNN | TNPO1 | TOP2B | TP53BP1 | TP63 | TPM3 | TPR | TRAF5 | TRERF1 | TRIM24 |
| TRIM58 | TRIO | TRPC5 | TRRAP | TSHR | TSHZ2 | TSHZ3 | TTF1 | TTL | TUBA3C |
| TUBB3 | TUSC3 | TXNIP | TYMS | TYR | TYRP1 | U2AF1 | UBE2D2 | UBR5 | UGT1A1 |
| UMPS | UPF3B | USH2A | USP6 | USP8 | VDAC2 | VEZF1 | VIM | WASF3 | WDR90 |
| WDTC1 | WHSC1 | WHSC1L1 | WIPF1 | WNK1 | WNT5A | WSCD2 | WWOX | WWP1 | WWP2 |
| XBP1 | XPC | XRCC1 | YBX1 | YY1AP1 | ZBTB16 | ZC3H11A | ZFP36L1 | ZFP36L2 | ZFPM2 |
| ZIC3 | ZNF217 | ZNF384 | ZNF521 | ZNF638 | ZNF750 | ZNF804B | ZNF814 | CD74 | EML4 |
| VEGFA | VHL | WT1 | XPO1 | XRCC2 | XRCC3 | ZFHX3 | ZMAT3 | SLC34A2 | PPARG |
| NCOA4 |  |  |  |  |  |  |  |  |  |
