# Supplementary material for: Association of emergence of new mutations in circulating tumuor DNA during chemotherapy with clinical outcome in metastatic colorectal cancer
Source: BMC Cancer. 2021 Jul 22;21:845. doi: 10.1186/s12885-021-08309-2 (PMC8296534; doi:10.1186/s12885-021-08309-2)
Supplement: Supplementary file 2 — Additional file 2 : Table S2. Abundance of trunk clone and its variations between baseline and after 4 cycles of chemotherapy. [file 12885_2021_8309_MOESM2_ESM.docx]

Table S2. Abundance of trunk clone and its variations between baseline and after 4 cycles of chemotherapy

| Patient ID | Somatic mutation | cHGVS | pHGVS | Function | Variant allele frequency | |
| --- | --- | --- | --- | --- | --- | --- |
|  |  |  |  |  | Baseline | Post-C4 |
| P01 | N.D. |  |  |  | N.D. | N.D. |
| P02 | TP53 | c.796G>A | p.G266R | missense | 6.4% | N.D. |
|  | KRAS | c.38G>A | p.G13D | missense | 3.6% | N.D. |
| P03 | APC | c.3830T>G | p.L1277* | nonsense | 45.5% | N.D. |
| P04 | TP53 | c.591G[2>1] | p.E198Kfs*49 | frameshift | 65.0% | 2.8% |
|  | APC | c.3925G>T | p.E1309* | nonsense | 32.1% | 1.3% |
| P05 | N.D. |  |  |  | N.D. | N.D. |
| P06 | TSC1 | c.428T[2>3] | p.P144Sfs*10 | frameshift | 67.5% | ^#^65.4% |
| P07 | TP53 | c.528C>A | p.C176* | nonsense | 11.4% | N.D. |
|  | KRAS | c.35G>C | p.G12A | missense | 5.8% | N.D. |
| P08 | TP53 | c.1024C>T | p.R342* | nonsense | 41.7% | 12.5% |
| P09 | PRRX1 | c.491T>C | p.L164P | missense | 4.0% | N.D. |
|  | KRAS | c.38G>A | p.G13D | missense | 3.3% | N.D. |
| P10 | RNF43 | c.620G>A | p.G207D | missense | 2.1% | N.D. |
|  | TP53 | c.524G>A | p.R175H | missense | 1.5% | N.D. |
| P11 | TP53 | c.596G>T | p.G199V | missense | 5.2% | 0.1% |
|  | APC | c.637C>T | p.R213* | nonsense | 4.6% | N.D. |
|  | BTK | c.100G>A | p.V34M | missense | 2.6% | 0.2% |
| P12 | N.D. |  |  |  | N.D. | N.D. |
| P13 | KRAS | c.38G>A | p.G13D | missense | 1.3% | N.D. |
|  | APC | c.2891T>G | p.L964* | nonsense | 1.2% | N.D. |
| P14 | KRAS | c.34G>A | p.G12S | missense | 33.6% | 4.7% |
|  | APC | c.4348C>T | p.R1450* | nonsense | 14.0% | 1.6% |
| P15 | KRAS | c.38G>A | p.G13D | missense | 73.2% | N/A |
| P16 | KRAS | c.436G>A | p.A146T | missense | 16.6% | 39.1% |
|  | APC | c.4132C>T | p.Q1378* | nonsense | 16.1% | 36.2% |
| P17 | TP53 | c.690_697del  CACCATCC | p.T231Lfs*6 | frameshift | 9.4% | 0.2% |
| P18 | RNF43 | c.391G>T | p.E131* | nonsense | 3.9% | 4.7% |
|  | BRAF | c.1799T>A | p.V600E | missense | 3.0% | 4.2% |
|  | TP53 | c.314G>T | p.G105V | missense | 2.8% | 3.6% |
| P19 | TP53 | c.701A>G | p.Y234C | missense | 80.1% | 22.1% |
| P20 | APC | c.4012C>T | p.Q1338* | nonsense | 38.7% | †10.4% |

cHGVS = Coding DNA reference sequences (Human Genome Variation Society)；pHGVS = Protein level amino acid sequences (Human Genome Variation Society)；N.D. = Not detected；N/A = Not available；# = Post-C3;† = Post-C5.
