# Supplementary figures and images for: Association of emergence of new mutations in circulating tumuor DNA during chemotherapy with clinical outcome in metastatic colorectal cancer
Source: BMC Cancer. 2021 Jul 22;21:845. doi: 10.1186/s12885-021-08309-2 (PMC8296534; doi:10.1186/s12885-021-08309-2)

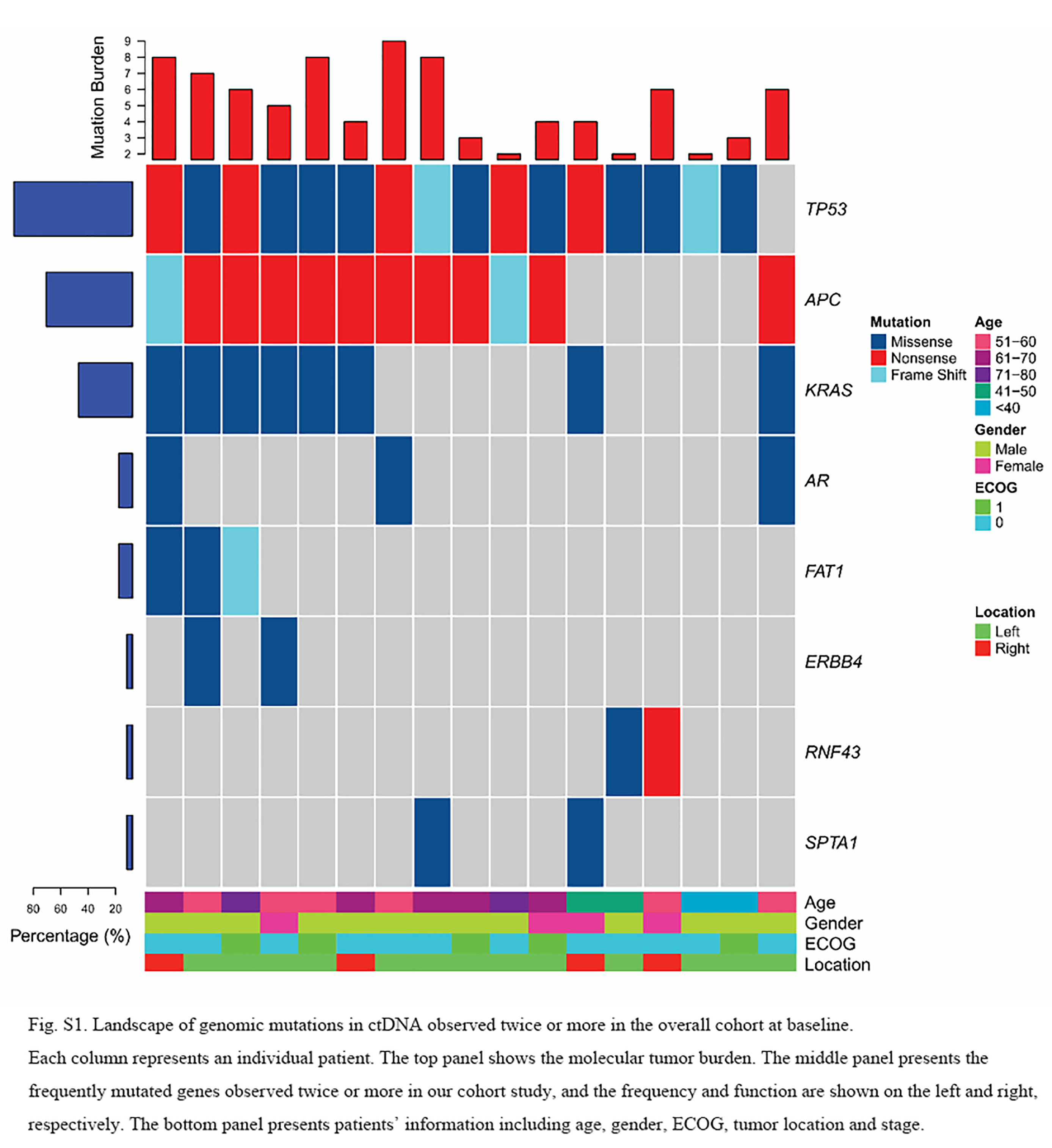

Supplement: Supplementary file 3 — Additional file 3 : Supplementary Fig. S1. Landscape of genomic mutations in ctDNA observed twice or more in the overall cohort at baseline. Each column represents an individual patient. The top panel shows the molecular tumor burden. The middle panel presents the frequently mutated genes observed twice or more in our cohort study, and the frequency and function are shown on the left and right, respectively. The bottom panel presents patients’ information including age, gender, ECOG, tumor location and stage. [file 12885_2021_8309_MOESM3_ESM.tif]

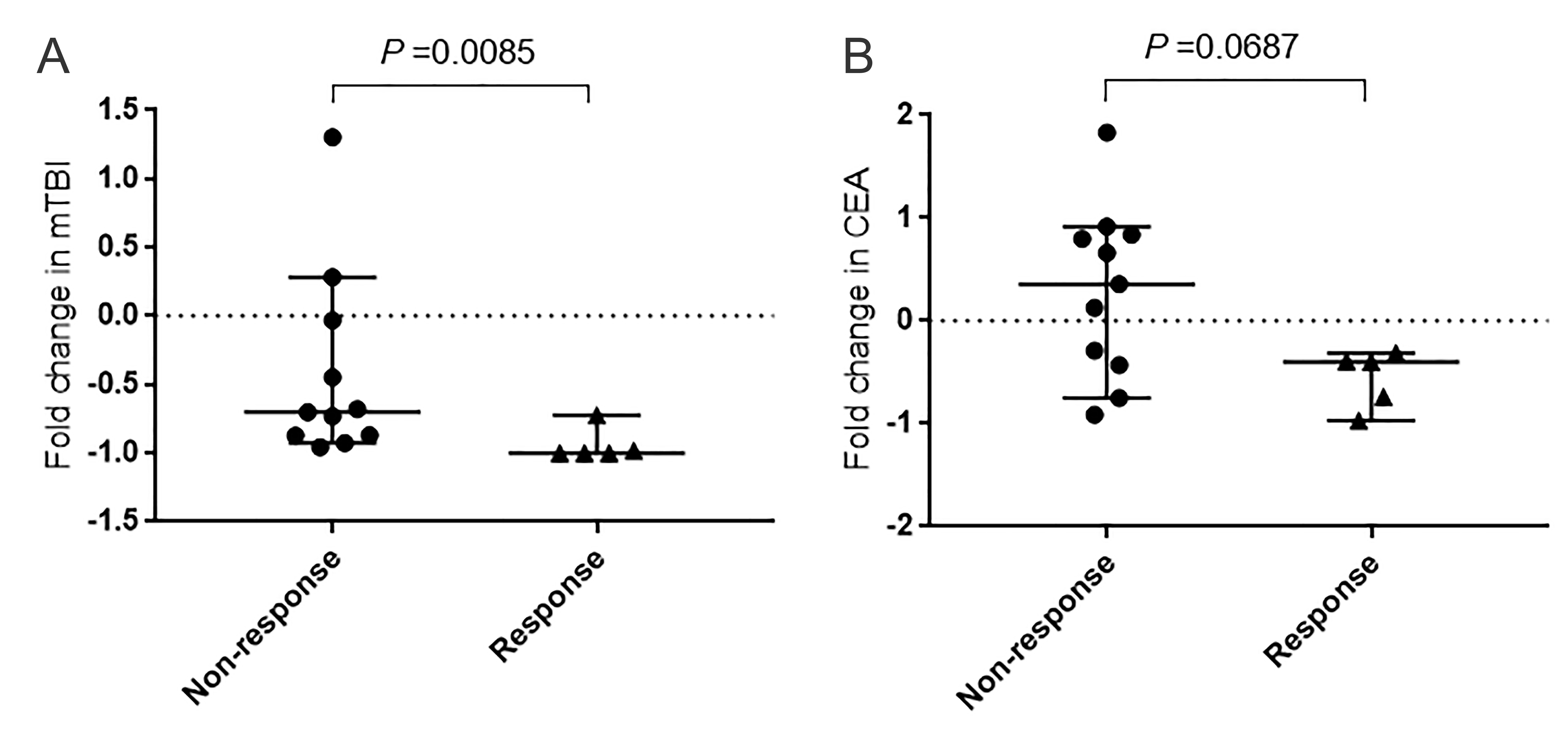

Supplement: Supplementary file 5 — Additional file 5 : Supplementary Fig. S2. The differences in fold changes in CEA and ctDNA between tumor response and non-response patients. A, Significant difference in fold change in mTBI after C4 between groups of patients with tumor response and non-response was observed: Mann–Whitney U-test at P = 0.0085. B, No significant difference was seen in fold change in serum CEA between groups of patients with tumor response and non-response: Mann–Whitney U-test at P = 0.0687. [file 12885_2021_8309_MOESM5_ESM.tif]
