# Supplementary material for: Association of emergence of new mutations in circulating tumuor DNA during chemotherapy with clinical outcome in metastatic colorectal cancer
Source: BMC Cancer. 2021 Jul 22;21:845. doi: 10.1186/s12885-021-08309-2 (PMC8296534; doi:10.1186/s12885-021-08309-2)
Supplement: Supplementary file 4 — Additional file 4 : Table S3. Abundances of ctDNA mutations and their variations between baseline and during treatment. [file 12885_2021_8309_MOESM4_ESM.docx]

Table S3**.** Abundances of ctDNA mutations and their variations between baseline and during treatment

| Patient ID | Time point | Somatic mutation | cHGVS | pHGVS | Function | VAFs (%) |
| --- | --- | --- | --- | --- | --- | --- |
| P01 | Baseline | N.D. |  |  |  | 0.00 |
| P01 | Post-C4 | N.D. |  |  |  | 0.00 |
| P02 | Baseline | TP53 | c.796G>A | p.G266R | missense | 6.10 |
| P02 | Baseline | APC | c.4348C>T | p.R1450* | nonsense | 3.70 |
| P02 | Baseline | KRAS | c.38G>A | p.G13D | missense | 3.50 |
| P02 | Baseline | FLT4 | c.434C>T | p.T145M | missense | 3.00 |
| P02 | Baseline | ERBB4 | c.632C>T | p.T211M | missense | 1.40 |
| P02 | Baseline | APC | c.1690C>T | p.R564* | nonsense | 1.20 |
| P02 | Baseline | FAT1 | c.7640A>T | p.Q2547L | missense | 0.90 |
| P02 | Post-C4 | N.D. |  |  |  | 0.00 |
| P03 | Baseline | APC | c.3830T>G | p.L1277* | nonsense | 44.30 |
| P03 | Baseline | TP53 | c.731G>A | p.G244D | missense | 40.10 |
| P03 | Baseline | EPHA3 | c.333G>T | p.L111F | missense | 1.70 |
| P03 | Post-C4 | N.D. |  |  |  | 0.00 |
| P03 | PD | APC | c.3830T>G | p.L1277* | nonsense | 35.90 |
| P03 | PD | TP53 | c.731G>A | p.G244D | missense | 36.00 |
| P03 | PD | OR2T4 | c.487C>T | p.R163C | missense | 23.00 |
| P03 | PD | BTK | c.481A>C | p.N161H | missense | 1.70 |
| P03 | PD | KRAS | c.35G>T | p.G12V | missense | 1.20 |
| P03 | PD | EPHA3 | c.333G>T | p.L111F | missense | 0.80 |
| P03 | PD | KRAS | c.34G>T | p.G12C | missense | 0.80 |
| P04 | Baseline | TP53 | c.591G[2>1] (std: c.591G[1] alt: c.592delG ) | p.E198Kfs*49 | frameshift | 65.80 |
| P04 | Baseline | IRS2 | c.2626C>T | p.R876C | missense | 37.70 |
| P04 | Baseline | APC | c.2365C>T | p.Q789* | nonsense | 35.40 |
| P04 | Baseline | APC | c.3925G>T | p.E1309* | nonsense | 32.20 |
| P04 | Baseline | NRAS | c.181C>A | p.Q61K | missense | 29.10 |
| P04 | Baseline | KDR | c.3728C>T | p.P1243L | missense | 6.30 |
| P04 | Baseline | AMOT | c.1022C>G | p.T341S | missense | 3.10 |
| P04 | Baseline | SPTA1 | c.2588G>C | p.G863A | missense | 0.70 |
| P04 | Post-C4 | IRS2 | c.2626C>T | p.R876C | missense | 3.40 |
| P04 | Post-C4 | NRAS | c.181C>A | p.Q61K | missense | 1.60 |
| P04 | Post-C4 | APC | c.3925G>T | p.E1309* | nonsense | 1.20 |
| P04 | Post-C4 | APC | c.2365C>T | p.Q789* | nonsense | 1.20 |
| P04 | Post-C4 | KDR | c.3728C>T | p.P1243L | missense | 0.96 |
| P04 | PD | TP53 | c.591G[2>1] (std: c.591G[1] alt: c.592delG ) | p.E198Kfs*49 | frameshift | 55.30 |
| P04 | PD | IRS2 | c.2626C>T | p.R876C | missense | 35.20 |
| P04 | PD | APC | c.2365C>T | p.Q789* | nonsense | 29.80 |
| P04 | PD | APC | c.3925G>T | p.E1309* | nonsense | 27.60 |
| P04 | PD | NRAS | c.181C>A | p.Q61K | missense | 25.18 |
| P04 | PD | KDR | c.3728C>T | p.P1243L | missense | 7.30 |
| P04 | PD | SPTA1 | c.2588G>C | p.G863A | missense | 1.40 |
| P05 | Baseline | N.D. |  |  |  | 0.00 |
| P05 | Post-C4 | N.D. |  |  |  | 0.00 |
| P06 | Baseline | MED12 | c.122T>G | p.V41G | missense | 80.40 |
| P06 | Baseline | TSC1 | c.428T[2>3] (std: c.429dupT alt: c.429_430insT ) | p.P144Sfs*10 | frameshift | 67.60 |
| P06 | Baseline | TP53 | c.734G>A | p.G245D | missense | 63.06 |
| P06 | Baseline | KRAS | c.35G>A | p.G12D | missense | 39.50 |
| P06 | Baseline | APC | c.3880C>T | p.Q1294* | nonsense | 40.50 |
| P06 | Baseline | PIK3R1 | c.1996G>A | p.E666K | missense | 20.60 |
| P06 | Baseline | APC | c.3184C>T | p.Q1062* | nonsense | 17.20 |
| P06 | Baseline | KIT | c.1438T>G | p.S480A | missense | 1.60 |
| P06 | Post-C2 | TP53 | c.734G>A | p.G245D | missense | 59.90 |
| P06 | Post-C2 | APC | c.3880C>T | p.Q1294* | nonsense | 45.60 |
| P06 | Post-C2 | TSC1 | c.428T[2>3] (std: c.429dupT alt: c.429_430insT ) | p.P144Sfs*10 | frameshift | 70.20 |
| P06 | Post-C2 | KRAS | c.35G>A | p.G12D | missense | 36.40 |
| P06 | Post-C2 | PIK3R1 | c.1996G>A | p.E666K | missense | 25.32 |
| P06 | Post-C2 | APC | c.3184C>T | p.Q1062* | nonsense | 19.40 |
| P06 | Post-C2 | KRAS | c.38G>A | p.G13D | missense | 0.36 |
| P06 | PD(post-C3) | MED12 | c.122T>G | p.V41G | missense | 76.36 |
| P06 | PD(post-C3) | TP53 | c.734G>A | p.G245D | missense | 57.80 |
| P06 | PD(post-C3) | APC | c.3880C>T | p.Q1294* | nonsense | 38.30 |
| P06 | PD(post-C3) | KRAS | c.35G>A | p.G12D | missense | 33.40 |
| P06 | PD(post-C3) | PIK3R1 | c.1996G>A | p.E666K | missense | 21.10 |
| P06 | PD(post-C3) | APC | c.3184C>T | p.Q1062* | nonsense | 18.50 |
| P07 | Baseline | TP53 | c.528C>A | p.C176* | nonsense | 11.30 |
| P07 | Baseline | KRAS | c.35G>C | p.G12A | missense | 5.00 |
| P07 | Baseline | APC | c.3916G>T | p.E1306* | nonsense | 3.20 |
| P07 | Baseline | DNMT3A | c.1817G>A | p.W606* | nonsense | 2.30 |
| P07 | Baseline | DNMT3A | c.526T>C | p.Y176H | missense | 2.59 |
| P07 | Baseline | FAT1 | c.4042A[2>1] | p.K1348Sfs*20 | frameshift | 0.87 |
| P07 | Post-C4 | DNMT3A | c.526T>C | p.Y176H | missense | 2.52 |
| P07 | Post-C4 | DNMT3A | c.1817G>A | p.W606* | nonsense | 2.10 |
| P07 | Post-C4 | CDK13 | c.604_605insT | p.R202Lfs*68 | frameshift | 1.10 |
| P07 | Post-C4 | MTOR | c.617G>A | p.R206H | missense | 0.73 |
| P07 | Post-C4 | FAT1 | c.4042A[2>1] | p.K1348Sfs*20 | frameshift | 0.78 |
| P08 | Baseline | TP53 | c.1024C>T | p.R342* | nonsense | 38.90 |
| P08 | Baseline | APC | c.3922AAAGA[2>1] | p.E1309Dfs*4 | frameshift | 33.68 |
| P08 | Post-C4 | TP53 | c.1024C>T | p.R342* | nonsense | 13.23 |
| P08 | Post-C4 | APC | c.3922AAAGA[2>1] | p.E1309Dfs*4 | frameshift | 9.20 |
| P08 | Post-C4 | BRCA2 | c.6232G>A | p.G2078R | missense | 0.90 |
| P08 | Post-C4 | MYC | c.1211A>G | p.K404R | missense | 0.32 |
| P08 | Post-C4 | FGFR2 | c.269A>T | p.E90V | missense | 0.56 |
| P08 | Post-C4 | EGFR | c.2327G>A | p.R776H | missense | 0.25 |
| P09 | Baseline | PRRX1 | c.491T>C | p.L164P | missense | 3.40 |
| P09 | Baseline | KRAS | c.38G>A | p.G13D | missense | 2.90 |
| P09 | Baseline | SPTA1 | c.2615C>T | p.S872F | missense | 1.50 |
| P09 | Baseline | TP53 | c.574C>T | p.Q192* | nonsense | 1.70 |
| P09 | Post-C4 | N.D. |  |  |  | 0.00 |
| P10 | Baseline | RNF43 | c.620G>A | p.G207D | missense | 2.10 |
| P10 | Baseline | TP53 | c.524G>A | p.R175H | missense | 1.40 |
| P10 | Post-C4 | PIK3R2 | c.451C>T | p.P151S | missense | 1.23 |
| P10 | Post-C4 | DNMT3A | c.2077C>T | p.R693C | missense | 0.57 |
| P10 | Post-C4 | NTRK1 | c.1859G>A | p.C620Y | missense | 0.60 |
| P10 | Post-C4 | MLL | c.10480A>G | p.N3494D | missense | 0.60 |
| P11 | Baseline | TP53 | c.596G>T | p.G199V | missense | 5.00 |
| P11 | Baseline | APC | c.637C>T | p.R213* | nonsense | 4.87 |
| P11 | Baseline | BTK | c.100G>A | p.V34M | missense | 2.69 |
| P11 | Baseline | APC | c.3883G>T | p.E1295* | nonsense | 2.30 |
| P11 | Post-C4 | N.D. |  |  |  | 0.00 |
| P12 | Baseline | N.D. |  |  |  | 0.00 |
| P12 | Post-C4 | N.D. |  |  |  | 0.00 |
| P13 | Baseline | KRAS | c.38G>A | p.G13D | missense | 1.20 |
| P13 | Baseline | AR | c.1670A>C | p.Q557P | missense | 1.10 |
| P13 | Baseline | APC | c.2891T>G | p.L964* | nonsense | 0.90 |
| P13 | Baseline | CBL | c.2267C>T | p.A756V | missense | 0.60 |
| P13 | Baseline | INHBA | c.1258G>A | p.V420M | missense | 0.67 |
| P13 | Baseline | CDKN2B | c.349G>A | p.V117M | missense | 0.54 |
| P13 | Post-C4 | N.D. |  |  |  | 0.00 |
| P14 | Baseline | KRAS | c.34G>A | p.G12S | missense | 31.69 |
| P14 | Baseline | TP53 | c.844C>T | p.R282W | missense | 20.30 |
| P14 | Baseline | APC | c.4348C>T | p.R1450* | nonsense | 12.30 |
| P14 | Baseline | APC | c.2805C>A | p.Y935* | nonsense | 9.60 |
| P14 | Post-C4 | KRAS | c.34G>A | p.G12S | missense | 4.80 |
| P14 | Post-C4 | TP53 | c.844C>T | p.R282W | missense | 1.90 |
| P14 | Post-C4 | APC | c.4348C>T | p.R1450* | nonsense | 1.50 |
| P14 | Post-C4 | APC | c.2805C>A | p.Y935* | nonsense | 0.90 |
| P15 | Baseline | KRAS | c.38G>A | p.G13D | missense | 73.20 |
| P15 | Baseline | TP53 | c.637C>T | p.R213* | nonsense | 69.10 |
| P15 | Baseline | CDK12 | c.1204A>C | p.K402Q | missense | 54.10 |
| P15 | Baseline | AR | c.1670A>C | p.Q557P | missense | 52.80 |
| P15 | Baseline | NTRK3 | c.505C>T | p.R169C | missense | 37.80 |
| P15 | Baseline | FAT1 | c.6544T>C | p.F2182L | missense | 32.80 |
| P15 | Baseline | APC | c.637C>T | p.R213* | nonsense | 30.00 |
| P15 | Baseline | APC | c.4661A[6>7] | p.T1556Nfs*3 | frameshift | 39.91 |
| P15 | Post-C4 | N/A |  |  |  |  |
| P16 | Baseline | KRAS | c.436G>A | p.A146T | missense | 18.30 |
| P16 | Baseline | APC | c.4132C>T | p.Q1378* | nonsense | 16.20 |
| P16 | Baseline | TP53 | c.658T>C | p.Y220H | missense | 8.20 |
| P16 | Baseline | ERBB4 | c.3668A>C | p.K1223T | missense | 1.80 |
| P16 | Baseline | BRCA2 | c.7582G>T | p.G2528* | nonsense | 1.10 |
| P16 | Post-C4 | KRAS | c.436G>A | p.A146T | missense | 42.00 |
| P16 | Post-C4 | APC | c.4132C>T | p.Q1378* | nonsense | 35.00 |
| P16 | Post-C4 | TP53 | c.658T>C | p.Y220H | missense | 25.90 |
| P16 | Post-C4 | COL25A1 | c.1090C>T | p.R364W | missense | 3.40 |
| P17 | Baseline | ACIN1 | c.1375G>A | p.A459T | missense | 7.50 |
| P17 | Baseline | TP53 | c.690_697delCACCATCC | p.T231Lfs*6 | frameshift | 9.40 |
| P17 | Post-C4 | N.D. |  |  |  | 0.00 |
| P18 | Baseline | RNF43 | c.391G>T | p.E131* | nonsense | 3.80 |
| P18 | Baseline | BRAF | c.1799T>A | p.V600E | missense | 3.00 |
| P18 | Baseline | TP53 | c.314G>T | p.G105V | missense | 2.60 |
| P18 | Baseline | PTCH1 | c.2098C>T | p.Q700* | nonsense | 2.10 |
| P18 | Baseline | ESR1 | c.190G>A | p.A64T | missense | 2.10 |
| P18 | Baseline | PCNXL2 | c.5275G>A | p.E1759K | missense | 2.00 |
| P18 | Baseline | TNN | c.2221C>T | p.R741C | missense | 0.71 |
| P18 | Post-C4 | RNF43 | c.391G>T | p.E131* | nonsense | 4.70 |
| P18 | Post-C4 | BRAF | c.1799T>A | p.V600E | missense | 4.10 |
| P18 | Post-C4 | TP53 | c.314G>T | p.G105V | missense | 3.20 |
| P18 | Post-C4 | ESR1 | c.190G>A | p.A64T | missense | 3.16 |
| P18 | Post-C4 | PTCH1 | c.2098C>T | p.Q700* | nonsense | 2.50 |
| P18 | Post-C4 | PCNXL2 | c.5275G>A | p.E1759K | missense | 2.10 |
| P19 | Baseline | TP53 | c.701A>G | p.Y234C | missense | 78.96 |
| P19 | Baseline | SMO | c.2117A>C | p.Q706P | missense | 23.13 |
| P19 | Baseline | EFCAB7 | c.939A>C | p.Q313H | missense | 22.21 |
| P19 | Post-C4 | TP53 | c.701A>G | p.Y234C | missense | 21.60 |
| P19 | Post-C4 | EFCAB7 | c.939A>C | p.Q313H | missense | 4.30 |
| P19 | Post-C4 | SMO | c.2117A>C | p.Q706P | missense | 4.70 |
| P19 | Post-C4 | MSH2 | c.2318A>T | p.K773M | missense | 1.40 |
| P19 | Post-C4 | NUP205 | c.445T>G | p.L149V | missense | 1.30 |
| P19 | Post-C4 | NOTCH1 | c.2809T>G | p.F937V | missense | 0.80 |
| P19 | Post-C4 | APC | c.3462AGA[3>2] | p.E1154[4>3] | cds-del | 2.78 |
| P20 | Baseline | TP53 | c.916C>T | p.R306* | nonsense | 45.60 |
| P20 | Baseline | APC | c.4012C>T | p.Q1338* | nonsense | 39.95 |
| P20 | Baseline | APC | c.694C>T | p.R232* | nonsense | 24.82 |
| P20 | Baseline | TP53 | c.892G>T | p.E298* | nonsense | 22.50 |
| P20 | Baseline | AR | c.2481C>A | p.F827L | missense | 21.10 |
| P20 | Baseline | ZAP70 | c.1614G>T | p.K538N | missense | 11.43 |
| P20 | Baseline | RICTOR | c.141A>T | p.R47S | missense | 8.90 |
| P20 | Baseline | PIK3CA | c.3140A>G | p.H1047R | missense | 7.30 |
| P20 | Baseline | MTOR | c.2491T>C | p.S831P | missense | 4.90 |
| P20 | Post-C5 | TP53 | c.916C>T | p.R306* | nonsense | 11.40 |
| P20 | Post-C5 | APC | c.4012C>T | p.Q1338* | nonsense | 10.40 |
| P20 | Post-C5 | AR | c.2481C>A | p.F827L | missense | 9.60 |
| P20 | Post-C5 | TP53 | c.892G>T | p.E298* | nonsense | 6.20 |
| P20 | Post-C5 | APC | c.694C>T | p.R232* | nonsense | 5.50 |
| P20 | Post-C5 | RICTOR | c.141A>T | p.R47S | missense | 2.30 |
| P20 | Post-C5 | PIK3CA | c.3140A>G | p.H1047R | missense | 1.20 |

cHGVS = Coding DNA reference sequences (Human Genome Variation Society); pHGVS = Protein level amino acid sequences (Human Genome Variation Society); N.D. = Not detected; N/A = Not available.
