# Supplementary material for: Association of emergence of new mutations in circulating tumuor DNA during chemotherapy with clinical outcome in metastatic colorectal cancer
Source: BMC Cancer. 2021 Jul 22;21:845. doi: 10.1186/s12885-021-08309-2 (PMC8296534; doi:10.1186/s12885-021-08309-2)
Supplement: Supplementary file 6 — Additional file 6 : Table S4. Identification of gene status in tumour tissue and baseline ctDNA. [file 12885_2021_8309_MOESM6_ESM.docx]

Table S4**.** Identification of gene status in tumour tissue and baseline ctDNA

| Patient ID  (N=10) | Gene status in tumour tissue | Mutant genes in ctDNA |
| --- | --- | --- |
| P03 | ARMS: KRAS WT, NRAS WT, HRAS WT  RT PCR: BRAF WT | APC, EPHA3, TP53 |
| P05 | NGS: PIK3CA MT, TP53 MT, KRAS MT  RT PCR: BRAF WT | Unidentified |
| P06 | NGS: APC MT, KRAS MT, TP53 MT  RT PCR: BRAF WT | APC, KRAS, TP53, TSC1, MED12, KIT, PIK3R1 |
| P07 | NGS: APC MT, KRAS MT, TP53 MT  RT PCR: BRAF WT | APC, KRAS, TP53, DNMT3A, FAT1 |
| P08 | ARMS: KRAS WT, NRAS WT | TP53, APC |
| P10 | ARMS: KRAS WT, NRAS WT, HRAS WT | RNF43, TP53 |
| P14 | ARMS: KRAS MT | KRAS, TP53, APC |
| P15 | NGS: KRAS MT | KRAS, TP53, CDK12, AR, NTRK3, FAT1, APC |
| P18 | NGS: BRAF MT, TP53 MT  ARMS: KRAS WT  RT PCR: BRAF | BRAF, TP53, RNF43 ,PTCH1, ESR1, TNN, PCNXL2 |
| P19 | ARMS: KRAS WT  RT PCR: BRAF WT | TP53, SMO, EFCAB7 |

ARMS = amplification refractory mutation system; RT PCR = real-time polymerase chain reaction; NGS = next generation sequencing; MT = mutant type; WT = wild type.
