# Supplementary material for: Association of emergence of new mutations in circulating tumuor DNA during chemotherapy with clinical outcome in metastatic colorectal cancer
Source: BMC Cancer. 2021 Jul 22;21:845. doi: 10.1186/s12885-021-08309-2 (PMC8296534; doi:10.1186/s12885-021-08309-2)
Supplement: Supplementary file 7 — Additional file 7 : Table S5: Multivariate Cox model assessing the impact on progression free survival of ctDNA variations observed between baseline and post-C4 after adjustment on the following variables: age, gender, Eastern Cooperative Oncology Group Performance Status, location of primary tumor and synchronicity of metastatic disease. [file 12885_2021_8309_MOESM7_ESM.docx]

Table S5: Multivariate Cox model assessing the impact on progression free survival of ctDNA variations observed between baseline and post-C4 after adjustment on the following variables: age, gender, Eastern Cooperative Oncology Group Performance Status, location of primary tumor and synchronicity of metastatic disease

|  | Progression Free Survival | | |
| --- | --- | --- | --- |
| Variable | Hazard Ratio | 95% CI | P value |
| Fold change in CEA | 0.30 | 0.04-2.16 | 0.2299 |
| Fold change in mTBI | 27.27 | 1.49-498.10 | 0.0257 |
